# Supplementary material for: Single-cell transcriptomics unveils molecular signatures of neuronal vulnerability in a mouse model of prion disease that overlap with Alzheimer’s disease
Source: Nat Commun. 2024 Nov 23;15:10174. doi: 10.1038/s41467-024-54579-2 (PMC11585576; doi:10.1038/s41467-024-54579-2)
Supplement: Supplementary file 1 — Supplementary Information [file 41467_2024_54579_MOESM1_ESM.docx]

Single-cell transcriptomics unveils molecular signatures of neuronal vulnerability in a mouse model of prion disease that overlap with Alzheimer’s disease

Supplementary information


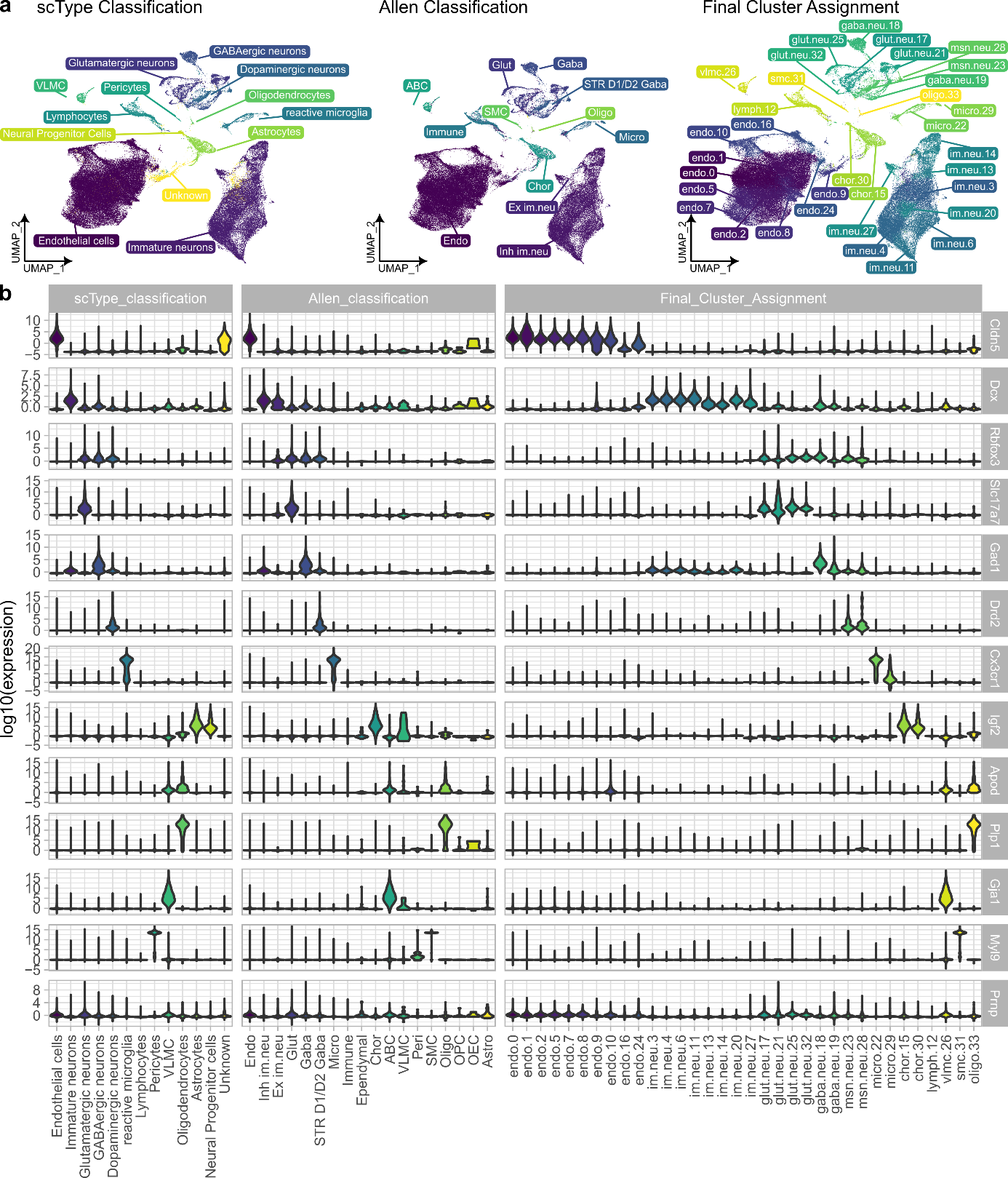


**Supplementary Figure 1. Cell type classification of individual clusters from the full transcriptional atlas (Related to Figure 1).** Individual transcriptomes were classified by brain cell type using scType, and the Allen brain Atlas` MapMyCells. Cell classification and marker transcript expression were used to assign a cell type identity to each cluster. **(a)** UMAP projections of the full transcriptional atlas with individual cells colored by scType classification, Allen cell classification, and final cluster assignment. **(b)** Marker transcript expression verified the identity of cell classes and final cluster assignment, visualized as violin plots.


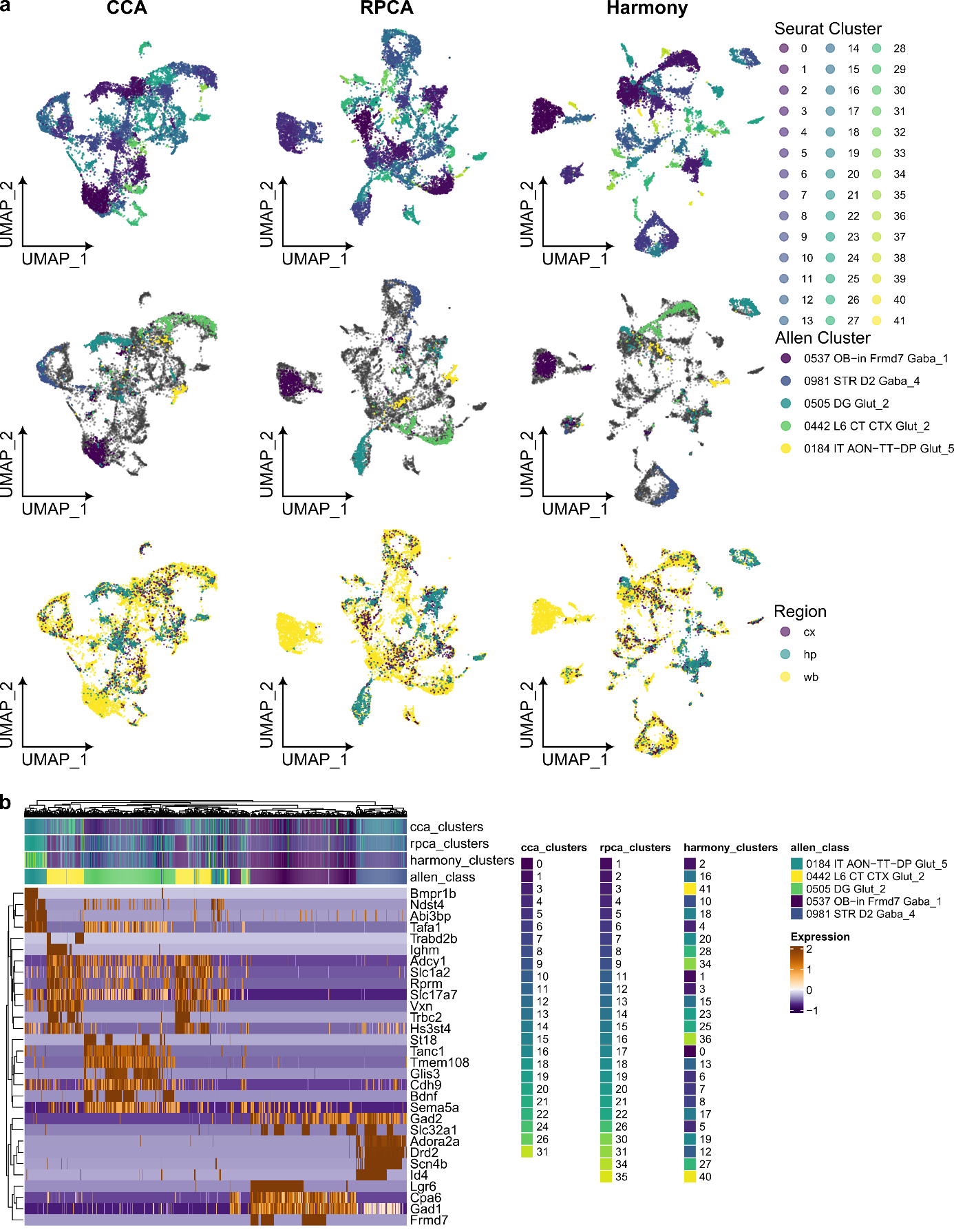


**Supplementary Figure 2. Comparison of CCA, RPCA, and Harmony methods for dataset integration and clustering (Related to Figure 2). (a)** UMAP plots show 10,754 high-quality neuronal transcriptomes isolated from 3 brain tissues, comparing clustering after dataset integration with Seurat’s CCA, RPCA, and Harmony methods. UMAP plots from each integration strategy are annotated with Seurat clusters, the top 5 allen brain atlas neuronal clusters identified within the dataset, and the brain region that the cells were isolated from. **(b)** Hierarchical clustering was used to examine gene expression of transcriptional markers of the top 5 Allen brain atlas neuronal clusters in relation to the integrated clusters assigned to each neuron.

**
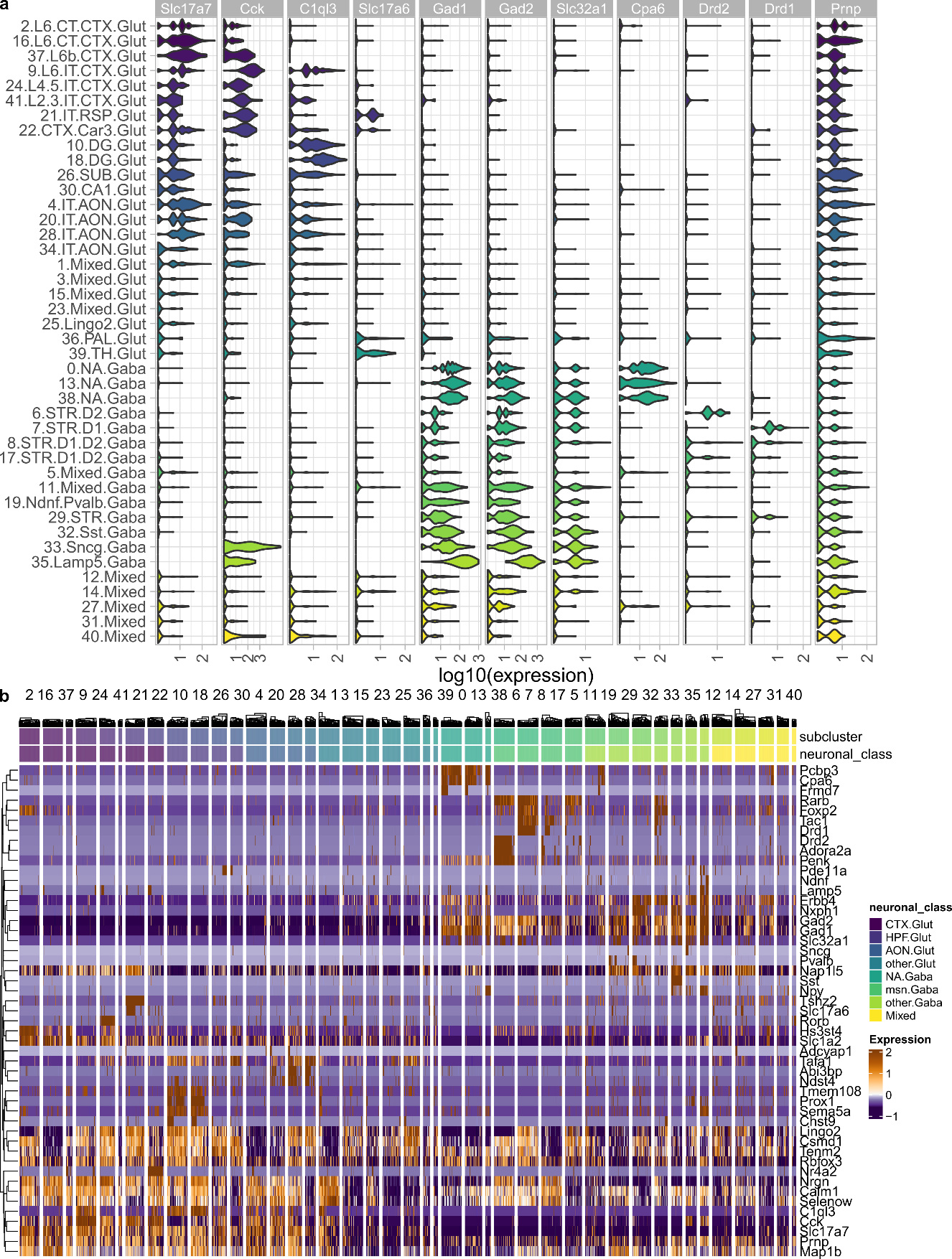
**

**Supplementary Figure 3. Expression of marker transcripts was used to confirm the identity of neuronal clusters (Related to Figure 2). (a)** Violin plot and **(b)** show expression of relevant transcriptional markers within each neuronal subcluster.

**
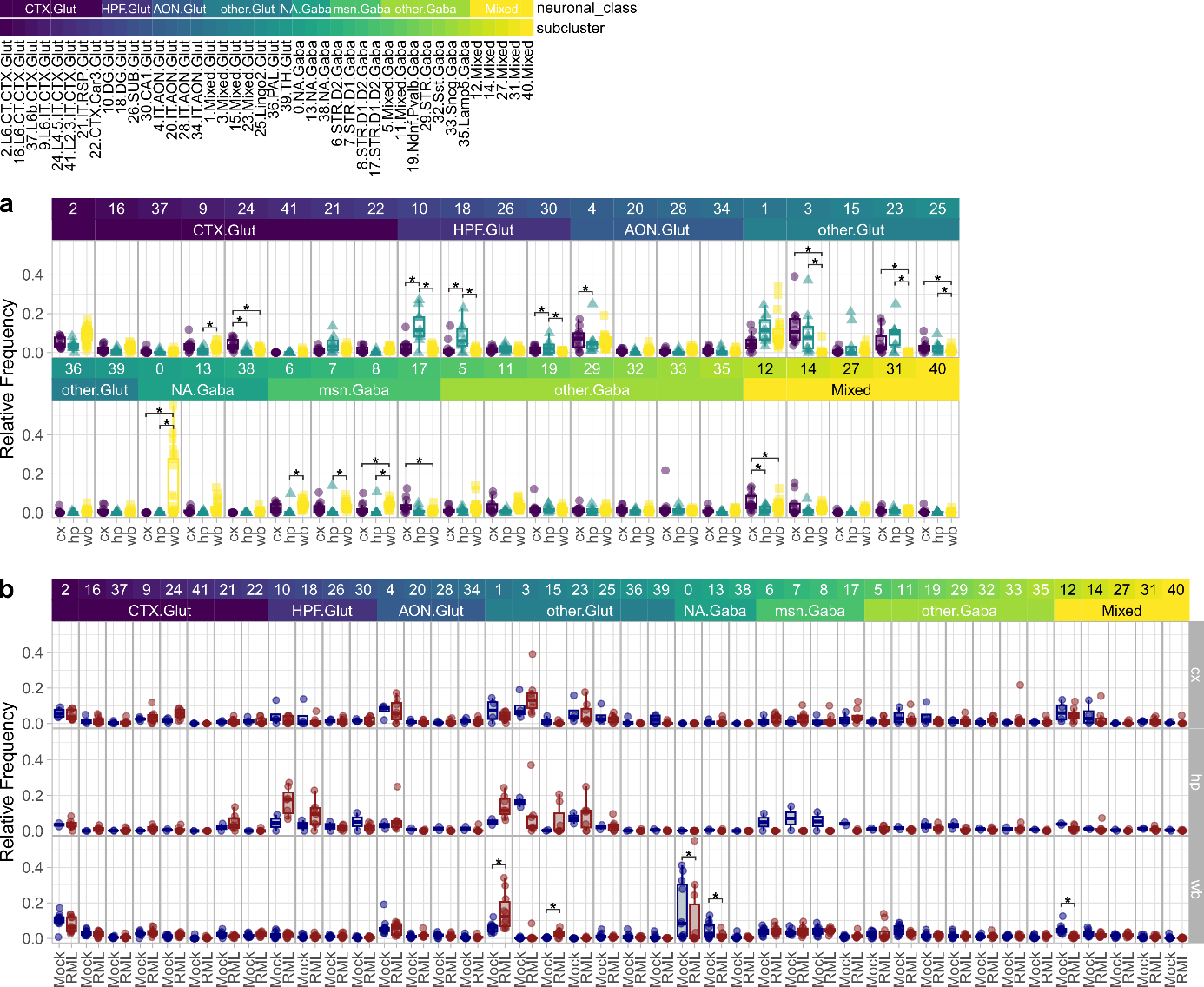
**

**Supplementary Figure 4. Extended analysis of cellular composition (Related to Figure 2). (a)** Neuronal subcluster composition was compared between each brain tissue used for cell isolation. **(b)** Neuronal subcluster composition was compared between prion (*n=5*) and mock (*n=4*) infected mice. Each brain tissue was analyzed separately. Credible changes in cell composition were identified using scCODA’s Bayesian model (* FDR < 0.05).


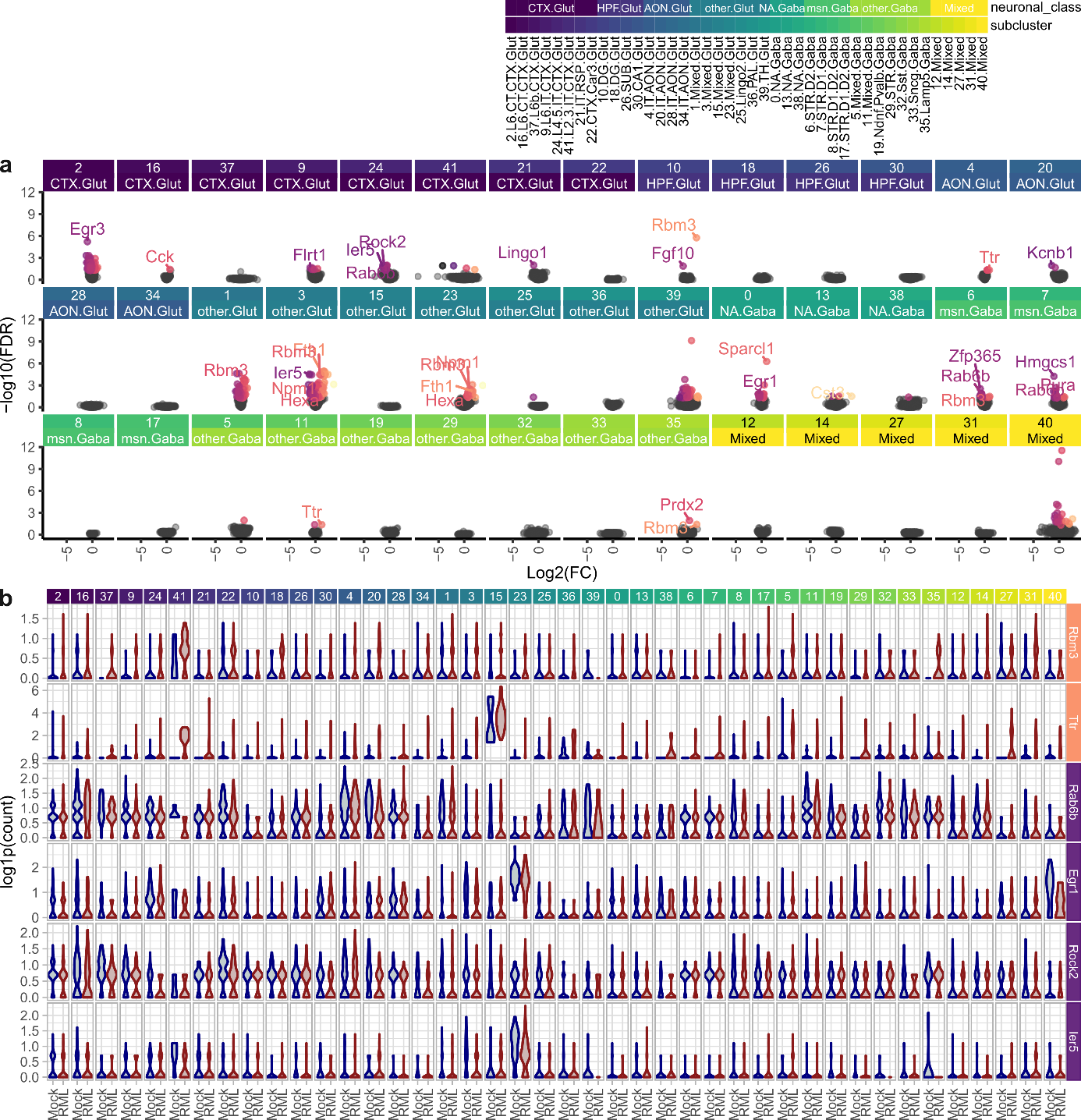


**Supplementary Figure 5. Extended analysis of prion-altered neuronal gene expression (Related to Figure 3). (a)** Volcano plots show prion-altered transcriptional changes identified among all neuronal subcluster using MAST’s two-part generalized linear model. **(b)** Violin plots show the prion-altered transcripts of select transcripts that were most consistent across neuronal subclusters.


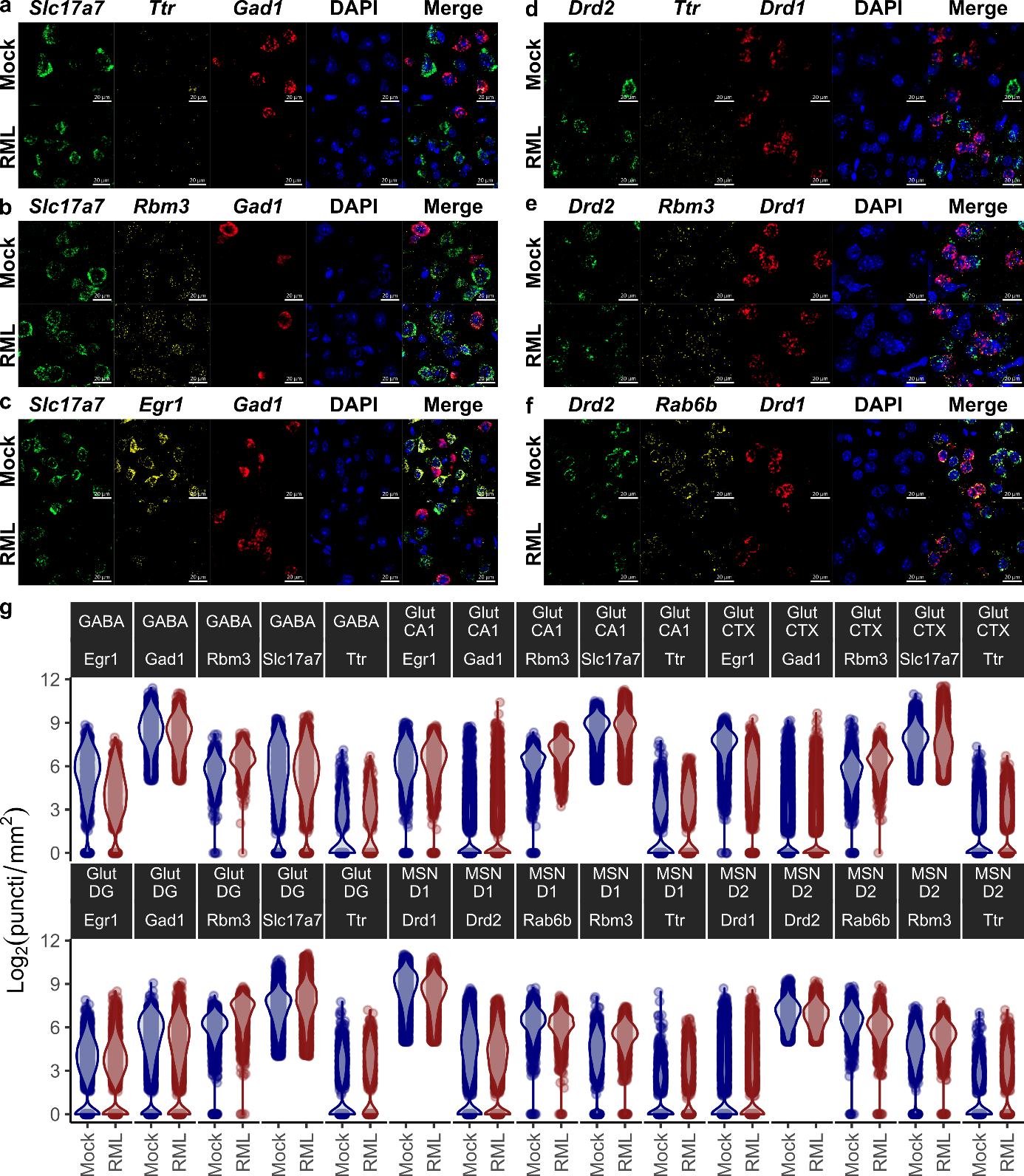


**Supplementary Figure 6. Extended RNA-FISH analysis of prion-altered neuronal gene expression (Related to Figure 4).** Representative images show RNAscope probe panels that were used to assess **(a)** *Ttr,* **(b)** *Rbm3*, and **(c)** *Egr1* expression in *Slc17a7*^+^ glutamatergic neurons and *Gad1*^+^ GABAergic neurons in the cortex, CA1, and dentate gyrus. Three additional RNAscrope probe panels assessed **(d)** *Ttr*, **(e)** *Rbm3*, and **(f)** *Rab6b* expression in *Drd1*^+^ and *Drd2*^+^ striatal medium spiny neurons. Scale bar = 20 µm. **(g)** Single-cell level gene expression was measured as probe puncti density and visualized in prion (*n=3*) and mock (*n=3*) infected mice.


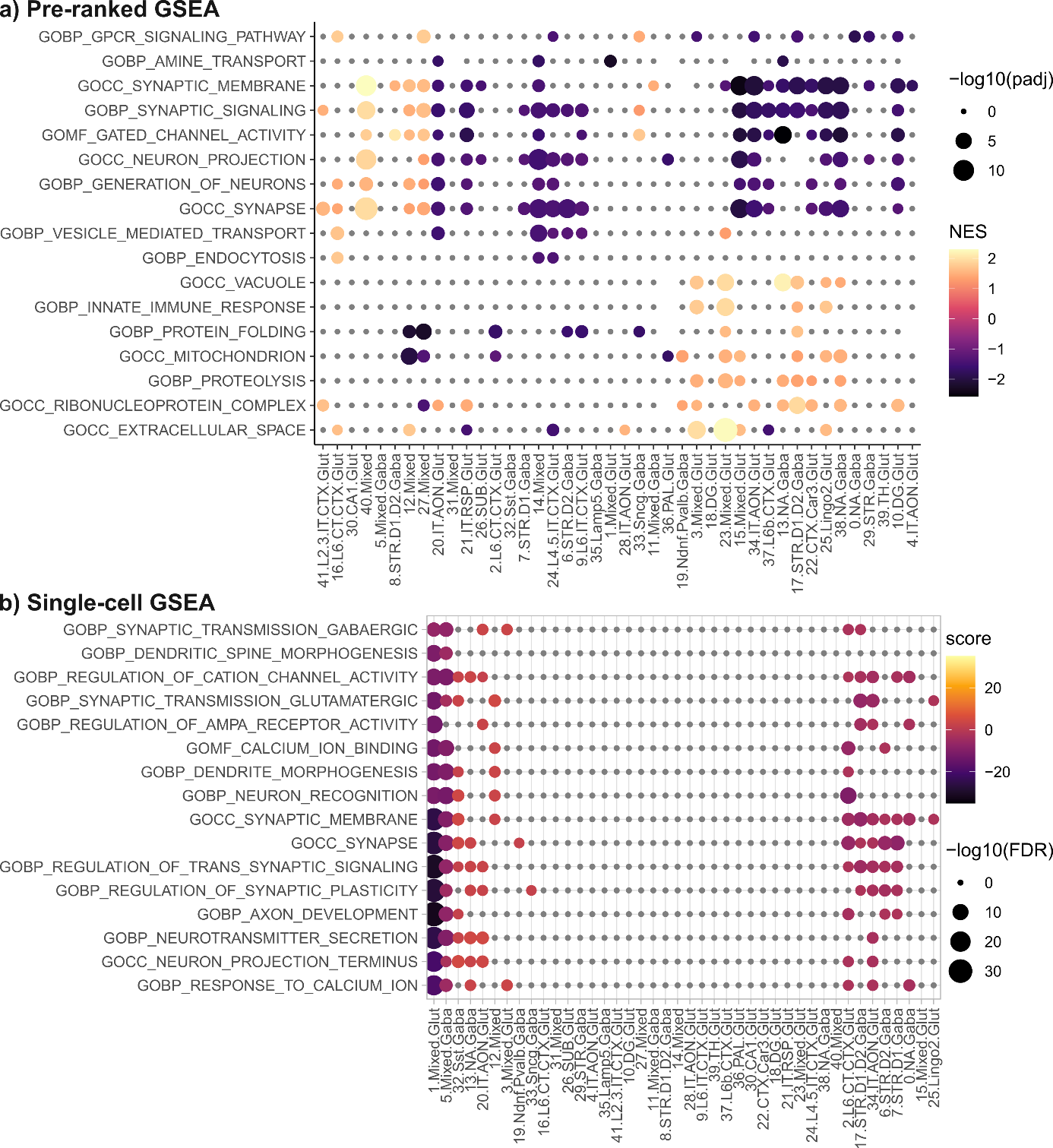


**Supplementary Figure 7. Extended Gene Set Enrichment Analysis (GSEA) of prion-altered gene expression within each neuronal cluster (Related to Figure 5). (a)** Prion-associated enrichment of pathways for each neuronal cluster were computed by applying GSEA (fgsea’s adaptive multi-level split Monte-Carlo scheme for *p*-value estimation) to pre-ranked prion-altered transcripts identified through differential expression analysis with MAST’s likelihood ratio test. **(b)** Enrichment of synapse-related gene sets was computed within individual cells using Escape, and compared between prion and mock-infected neurons within each cluster using Escape’s t-test. Prion-altered changes in single-cell synapse gene expression are summarized for each neuronal cluster.

**
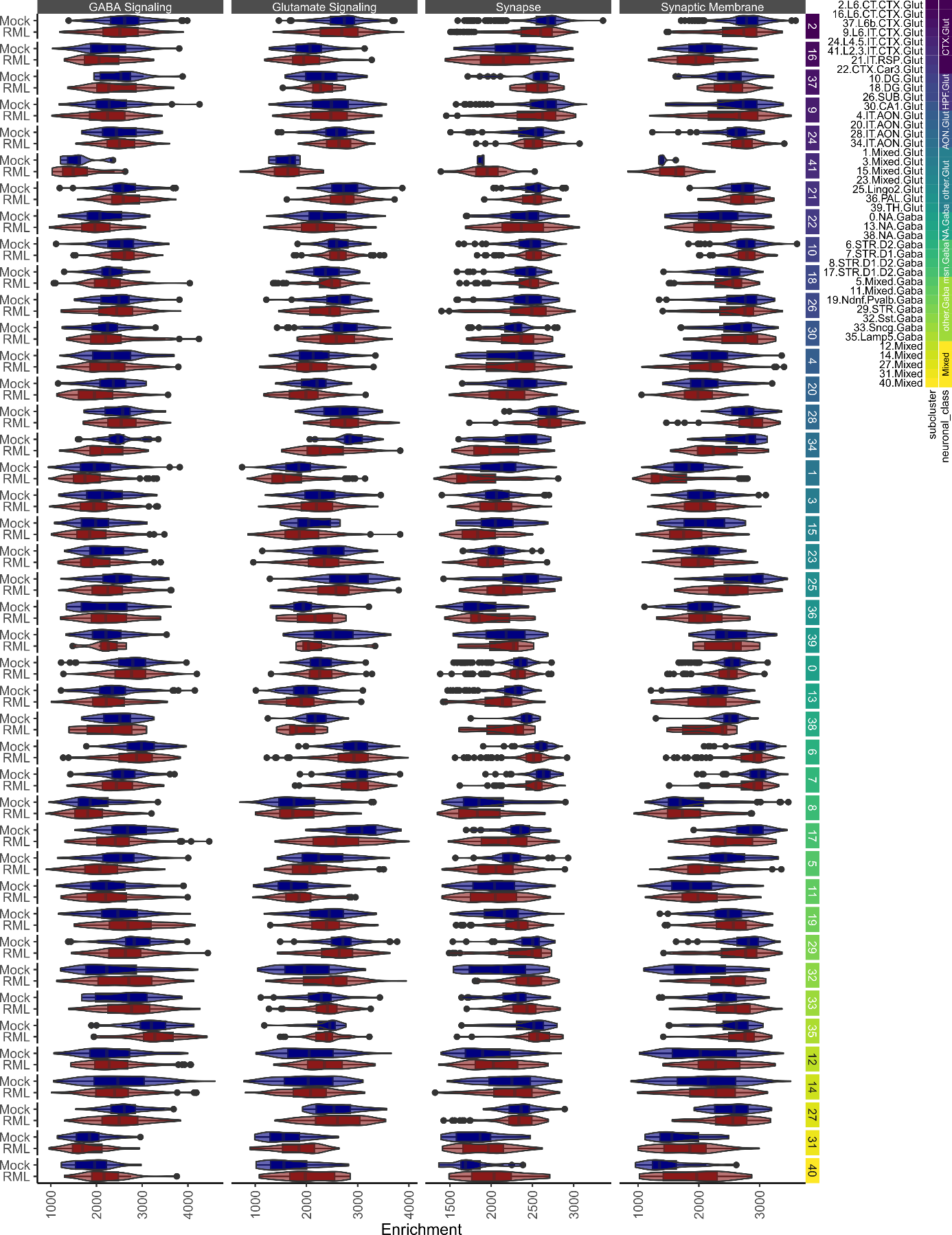
**

**Supplementary Figure 8. Neuronal single-cell gene-set enrichment of select synaptic gene sets (Related to Figure 5).** Enrichment of synapse-related gene sets was computed within individual cells using Escape. Distribution of single-cell gene-set enrichment is shown for select synapse-related gene sets across all neuronal sub-clusters.


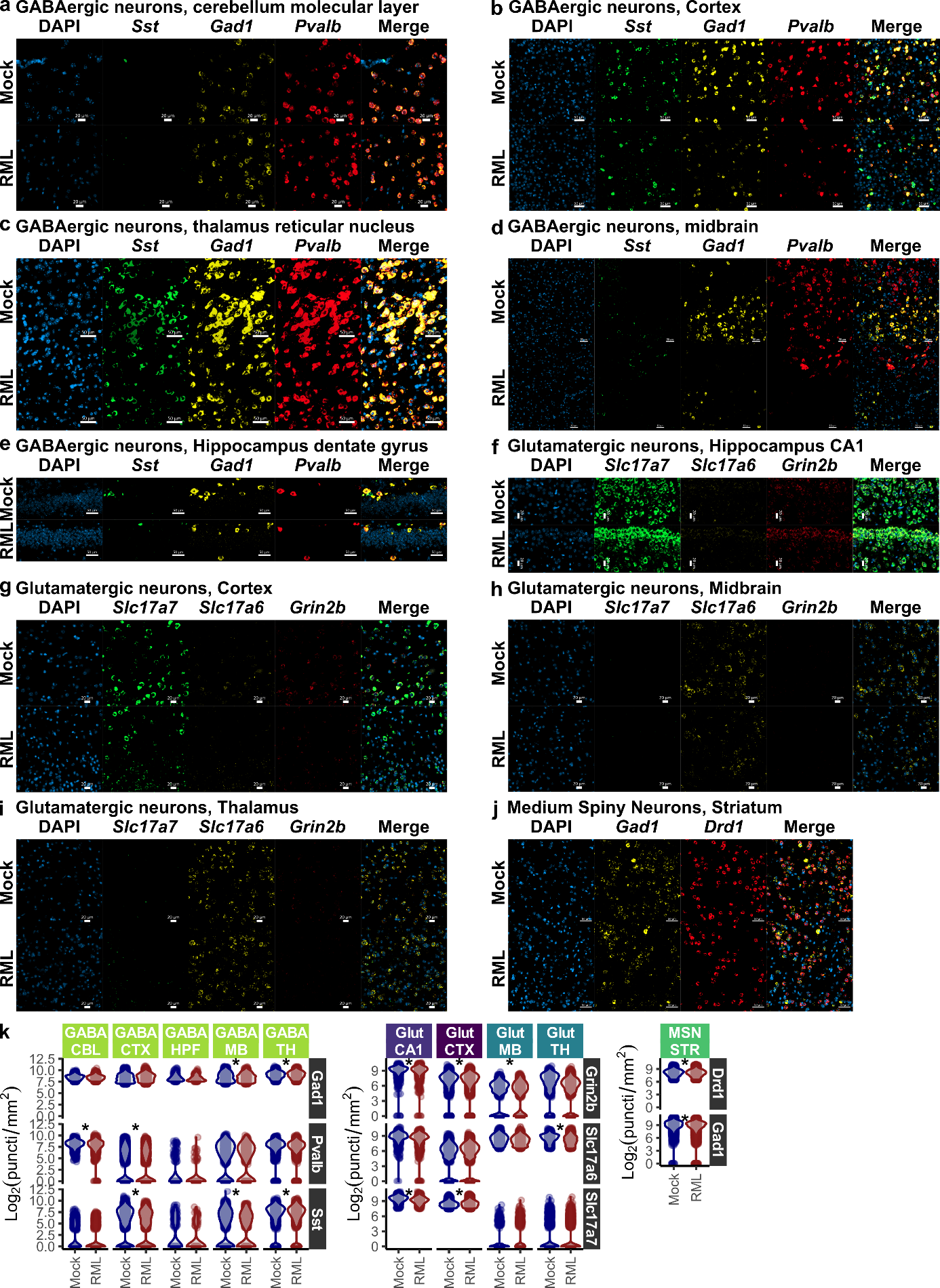


**Supplementary Figure 9. RNA-FISH-based assessment of neuronal vulnerability in prion infected mice (Related to Figure 6).** Formalin-fixed paraffin-embedded brain sections were stained with a panel of RNAscope probes targeting GABAergic neuronal markers *Sst*, *Gad1,* and *Pvalb,* Glutamatergic neuronal markers *Slc17a7, Slc17a6*, and *Grin2b*, and medium spiny neuronal markers *Gad1*, and *Drd1*. Sections were imaged with a scanning fluorescence microscope. Representative regions of interest (ROIs) taken from prion (RML) and mock-infected mice (*n=3*) are shown for: GABAergic neurons in the **(a)** cerebellum, **(b)** cortex, **(c)** thalamus, **(d)** midbrain, **(e)** hippocampus, Glutamatergic neurons in the **(f)** CA1, **(g)** cortex, **(h)** midbrain, **(i)** thalamus, and **(j)** striatal medium spiny neurons. **(k)** Neuronal transcript expression within each region, quantified via per-cell measurements of RNAscope-probe puncti density, was compared between prion- and mock-infected mice by fitting to prion disease status, mouse number, and ROI number with a general linear model, and applying the Bonferroni Hochberg false discovery correction. * FDR corrected p-value < 0.05.


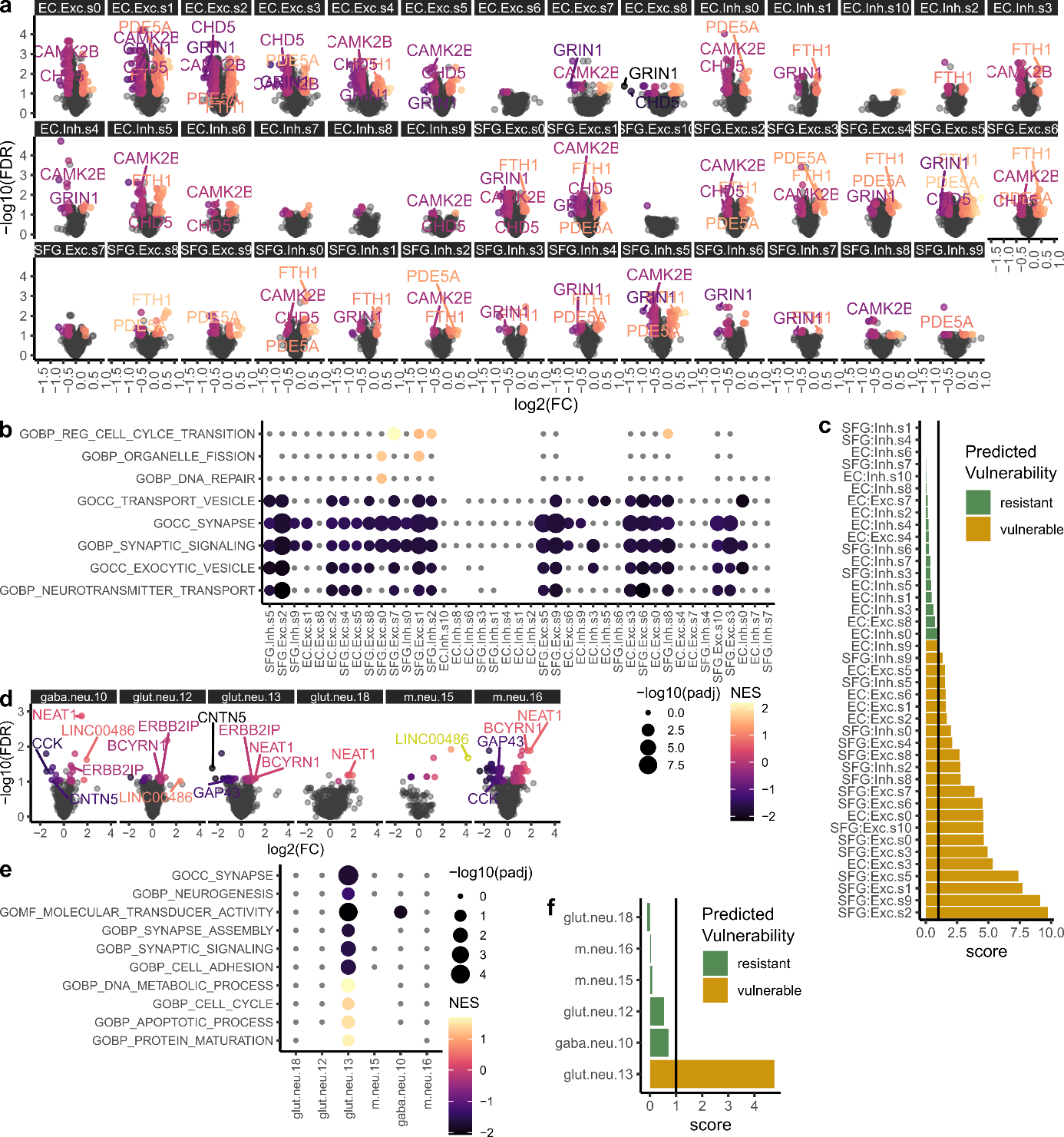


**Supplementary Figure 10. Characterization of gene expression and neuronal vulnerability in previously published snRNAseq datasets of Alzheimer’s disease (Related to Figure 8).** For comparison with prion disease, we applied our approach of assessing neuronal vulnerability to snRNAseq datasets of Alzheimer’s disease previously published by Grubman et al. and Leng et al. First, we employed MAST’s likelihood ratio test to identify transcipts that were differentially expressed in human Alzheimer’s disease cases, by **(a)** comparing between Braak Scores in the Leng dataset and **(d)** comparing AD vs controls in the Grubman dataset. Volcano plots summarize the differential expression results. Next, GSEA (fgsea’s adaptive multi-level split Monte-Carlo scheme for *p*-value estimation) was applied to the lists of differentially expressed transcripts, confirming that the decrease in synapse-related gene expression was the major signature of Alzheimer’s disease in both the **(b)** Leng and **(e)** Grubman datasets. Finally, we computed vulnerability scores as the –signed(-log10(FDR)) of AD-associated enrichment of the GOCC Synapse gene set for neuronal sub-clusters in the **(c)** Leng and **(f)** Grubman datasets. We retained the neuronal sub-cluster annotations as reported in the original studies. MAST’s likelihood ratio test was used to identify vulnerability-correlated transcripts based on differential expression against vulnerability score amongst neurons with Braak Score = 0 in the Leng dataset, and control neurons in the Grubman dataset (Shown in Figure 8).
