# Supplementary material for: Single-cell transcriptomics unveils molecular signatures of neuronal vulnerability in a mouse model of prion disease that overlap with Alzheimer’s disease
Source: Nat Commun. 2024 Nov 23;15:10174. doi: 10.1038/s41467-024-54579-2 (PMC11585576; doi:10.1038/s41467-024-54579-2)
Supplement: Supplementary file 2 — Description of Additional Supplementary Files [file 41467_2024_54579_MOESM2_ESM.docx]

Description of Additional Supplementary Information

Supplementary Data 1. Summary statistics for each scRNAseq library pre- and post-quality control.

Supplementary Data 2. Globally distinguishing cell cluster markers.

Supplementary Data 3. Cell composition analysis with scCODA.

Supplementary Data 4. Summary of all prion-altered genes identified through differential expression analysis between prion- and mock-infected cells.

Supplementary Data 5. Vulnerability markers that differed in expression between neurons classified as resistant and vulnerable to damage in prion and Alzheimer’s disease.

Supplementary Data 6. Summary of RNAscope probe panels with number of ROI images and individual cells profiled.
