# Supplementary material for: Single-cell transcriptomics unveils molecular signatures of neuronal vulnerability in a mouse model of prion disease that overlap with Alzheimer’s disease
Source: Nat Commun. 2024 Nov 23;15:10174. doi: 10.1038/s41467-024-54579-2 (PMC11585576; doi:10.1038/s41467-024-54579-2)
Supplement: Supplementary file 9 — Reporting Summary [file 41467_2024_54579_MOESM9_ESM.pdf]

nature portfolio

Corresponding author(s): Stephanie Booth

Last updated by author(s): Oct 22, 2024

Reporting Summary

Nature Portfolio wishes to improve the reproducibility of the work that we publish. This form provides structure for consistency and transparency in reporting. For further information on Nature Portfolio policies, see our Editorial Policies and the Editorial Policy Checklist.

Statistics

For all statistical analyses, confirm that the following items are present in the figure legend, table legend, main text, or Methods section.

n/a	Confirmed	
☐	☒	The exact sample size (*n*) for each experimental group/condition, given as a discrete number and unit of measurement
☒	☐	A statement on whether measurements were taken from distinct samples or whether the same sample was measured repeatedly
☐	☒	The statistical test(s) used AND whether they are one- or two-sided<br>*Only common tests should be described solely by name; describe more complex techniques in the Methods section.*
☒	☐	A description of all covariates tested
☐	☒	A description of any assumptions or corrections, such as tests of normality and adjustment for multiple comparisons
☐	☒	A full description of the statistical parameters including central tendency (e.g. means) or other basic estimates (e.g. regression coefficient) AND variation (e.g. standard deviation) or associated estimates of uncertainty (e.g. confidence intervals)
☐	☒	For null hypothesis testing, the test statistic (e.g. *F*, *t*, *r*) with confidence intervals, effect sizes, degrees of freedom and *P* value noted<br>*Give P values as exact values whenever suitable.*
☐	☒	For Bayesian analysis, information on the choice of priors and Markov chain Monte Carlo settings
☒	☐	For hierarchical and complex designs, identification of the appropriate level for tests and full reporting of outcomes
☐	☒	Estimates of effect sizes (e.g. Cohen's *d*, Pearson's *r*), indicating how they were calculated

Our web collection on statistics for biologists contains articles on many of the points above.

Software and code

Policy information about availability of computer code

Data collection	Fluorescence microscopy images for RNA-FISH were acquired and processed with Zeiss Zen Blue (v3.8) software.
Data analysis	Raw single-cell RNAseq data was pre-processed with cellranger (v7.1.0)<br>RNA FISH microscopy images were analyzed with CellProfiler (v4.2.5)<br>Downstream analysis of single-cell RNAseq and RNA-FISH data were done using custom R scripts in R (v4.3.1). The following R packages were used: Seurat (v4.3.0), soupX (v1.6.2), DoubletFinder (v2.0.3), SCType (v1.0), Enrichr (v3.2), Escape (v1.1.0), MAST (v1.26.0), fgsea (v1.26.0), ComplexHeatmap (v2.18.0), Dplyr (v1.1.2), and ggplot2 (v3.4.2).<br>Compositional analysis of scRNAseq data was done with scCODA (v0.1.9), using custom code in Python (v3.11).<br>Synaptic genes were analyzied with SynGo (v1.2).<br>All custom R and python code is available from the following GitHub repository: https://github.com/jslota/scRNAseq_prion_neuronal_vulnerability/

For manuscripts utilizing custom algorithms or software that are central to the research but not yet described in published literature, software must be made available to editors and reviewers. We strongly encourage code deposition in a community repository (e.g. GitHub). See the Nature Portfolio guidelines for submitting code & software for further information.

Data

Policy information about availability of data

All manuscripts must include a data availability statement. This statement should provide the following information, where applicable:

- Accession codes, unique identifiers, or web links for publicly available datasets
- A description of any restrictions on data availability
- For clinical datasets or third party data, please ensure that the statement adheres to our policy

All new single-cell RNA sequencing data generated by this study are available the Broad Institutes single cell portal as study # SCP2450 [https://singlecell.broadinstitute.org/single_cell/study/SCP2450/scrnaseq-neuronal-vulnerability-in-prion-disease]. Additionally, raw gene expression matrices for each individual library are available from Gene Expression Omnibus under accession number GSE249382 [https://www.ncbi.nlm.nih.gov/geo/query/acc.cgi?acc=GSE249382].

Research involving human participants, their data, or biological material

Policy information about studies with human participants or human data. See also policy information about sex, gender (identity/presentation), and sexual orientation and race, ethnicity and racism.

Reporting on sex and gender	No new human participants, data, or biological material were used in this study.
Reporting on race, ethnicity, or other socially relevant groupings	No new human participants, data, or biological material were used in this study.
Population characteristics	No new human participants, data, or biological material were used in this study.
Recruitment	No new human participants, data, or biological material were used in this study.
Ethics oversight	No new human participants, data, or biological material were used in this study.

Note that full information on the approval of the study protocol must also be provided in the manuscript.

Field-specific reporting

Please select the one below that is the best fit for your research. If you are not sure, read the appropriate sections before making your selection.

☒ Life sciences ☐ Behavioural & social sciences ☐ Ecological, evolutionary & environmental sciences

For a reference copy of the document with all sections, see nature.com/documents/nr-reporting-summary-flat.pdf

Life sciences study design

All studies must disclose on these points even when the disclosure is negative.

Sample size	We used 5 prion-infected and 4 mock-infected mice for single-cell RNA sequencing experiments. Only one mouse could be processed for scRNAseq per day, and sample size was also limited by the availability of reagents.<br>We used a separate set of 3 prion-infected and 3 mock-infected mice for validation with RNA FISH.
Data exclusions	No data were excluded from the analysis.
Replication	Findings from single-cell RNA sequencing data were validated using RNA FISH. All attempts of replication were successful and are shown in the manuscript.
Randomization	Mice were randomly allocated to the prion and mock treatment groups.
Blinding	Blinding could not be performed owing to the bio-hazardous nature of the work.

Reporting for specific materials, systems and methods

We require information from authors about some types of materials, experimental systems and methods used in many studies. Here, indicate whether each material, system or method listed is relevant to your study. If you are not sure if a list item applies to your research, read the appropriate section before selecting a response.

Materials & experimental systems

n/a	Involved in the study
☒	☐ Antibodies
☒	☐ Eukaryotic cell lines
☒	☐ Palaeontology and archaeology
☐	☒ Animals and other organisms
☒	☐ Clinical data
☒	☐ Dual use research of concern
☒	☐ Plants

Methods

n/a	Involved in the study
☒	☐ ChIP-seq
☒	☐ Flow cytometry
☒	☐ MRI-based neuroimaging

Animals and other research organisms

Policy information about studies involving animals; ARRIVE guidelines recommended for reporting animal research, and Sex and Gender in Research

Laboratory animals	This study utilized adult female CD1 mice (mus musculus) that were inoculated at 7 weeks of age. These mice originated from the University of Manitoba animal colony. Mice were housed with a light/dark cycle of 12/12 h at 21 °C and relative humidity of 50%.
Wild animals	This study did not involve wild animals.
Reporting on sex	Only female mice were used.
Field-collected samples	This study did not involve samples collected from the field.
Ethics oversight	The Animal Care Committee of the Canadian Science Centre for Human and Animal Health approved all animal experiments and procedures under animal use document (AUD) # H-20-024.

Note that full information on the approval of the study protocol must also be provided in the manuscript.

Plants

Seed stocks	*Report on the source of all seed stocks or other plant material used. If applicable, state the seed stock centre and catalogue number. If plant specimens were collected from the field, describe the collection location, date and sampling procedures.*
Novel plant genotypes	*Describe the methods by which all novel plant genotypes were produced. This includes those generated by transgenic approaches, gene editing, chemical/radiation-based mutagenesis and hybridization. For transgenic lines, describe the transformation method, the number of independent lines analyzed and the generation upon which experiments were performed. For gene-edited lines, describe the editor used, the endogenous sequence targeted for editing, the targeting guide RNA sequence (if applicable) and how the editor was applied.*
Authentication	*Describe any authentication procedures for each seed stock used or novel genotype generated. Describe any experiments used to assess the effect of a mutation and, where applicable, how potential secondary effects (e.g. second site T-DNA insertions, mosiacism, off-target gene editing) were examined.*
